# Two-year clinical and economic burden, risk and outcomes following application of software-assisted hexapod ring fixation systems

**DOI:** 10.1186/s12891-021-04934-x

**Published:** 2022-01-03

**Authors:** J. Spence Reid, Mollie Vanderkarr, Bidusee Ray, Abhishek Chitnis, Chantal E. Holy, Charisse Sparks

**Affiliations:** 1 Penn State Health, Hershey, PA USA; 2 DePuy Synthes, West Chester, PA USA; 3 Mu Sigma, Bengalore, India; 4 Real World Data Sciences, Medical Device Epidemiology, Johnson & Johnson, 410 George St, New Brunswick, NJ 08901 USA

**Keywords:** Long bone deformities, Hexapod ring fixation, Longitudinal, Complications, Resource utilization, Insurance payments, Ilizarov

## Abstract

**Background:**

Multiplanar external fixation systems that employ software-assisted deformity correction consist of rings connected by angled struts, defined as hexapod ring fixators (HRF). Costs and outcomes associated with the application of HRFs are not well documented. This study was designed to provide a nationwide baseline understanding of the clinical presentation, risks, outcomes and payer costs, and healthcare resource utilization (HCU) of patients requiring application of an HRF, from the day of, and up to 2 years, post-application.

**Methods:**

Patients with HRF application (“index”) between 2007 and 2019 within the IBM Marketscan® Commercial Claims database were identified and categorized based on diagnosis: acquired deformity, arthropathy, congenital deformity, deep infection, nonunion, fracture, and other post-operative fracture sequelae. Demographics, comorbidities at index, complications post-index, HCU, and payments were analyzed. Payments were estimated using a generalized linear model and were adjusted for inflation to the 2020 consumer price index. Rates of deep infection and amputation were estimated up to 2 years post-index using Poisson regressions, and risk factors for each were estimated using logistic regression models.

**Results:**

Six hundred ninety-five patients were included in our study (including 219 fractures, 168 congenital deformities, 68 deep infections, 103 acquired deformities). Comorbidities at index were significantly different across groups: less than 2% pediatrics vs 18% adults had 3 or more comorbidities, < 1% pediatric vs 29% adults had diabetes. Index payments ranged from $39,250–$75,350, with 12-months post-index payments ranging from $14,350 to $43,108. The duration of the HRF application ranged from 96 days to 174 days. Amputation was observed in patients with deep infection (8.9, 95% confidence interval (CI): 3.2–23.9%), nonunion (5.0, 95%CI: 1.6–15.4%) or fracture (2.7, 95%CI: 0.9–7.6%) at index. Complicated diabetes was the main predictor for deep infection (odds ratio (OR): 5.14, 95%CI: 2.50–10.54) and amputation (OR: 5.26, 95%CI: 1.79–15.51).

**Conclusions:**

Findings from this longitudinal analysis demonstrate the significant heterogeneity in patients treated with HRF, and the wide range in treatment intensity, payments, and outcomes. Risks for deep infection and amputation were primarily linked to the presence of complicated diabetes at the time of HRF application, suggesting a need for careful management of comorbid chronic conditions in patients requiring HRF for orthopedic care.

## Background

Computer-assisted hexapod ring fixation systems (HRF) are used for a wide variety of pathological conditions, including deformity correction, complex fractures, and post-traumatic complications [1–7]. These devices consist of external ring fixators and six connecting struts that allow precise simultaneous movement between the two main rings in 6 degrees of freedom. Computer software is needed to create the plan of movement of each strut due to the complexity of mathematics [5]. These devices are manually adjusted over the treatment period, to correct the deformities and/or lengthen/shorten bone segments. The generation of complex treatment plans is challenging as it requires significant clinical expertise, sophisticated techniques, multiple follow-up visits, and diagnostic imaging, resulting in radiation exposure to patients [8, 9].

Due to its complexity, it is not uncommon for patients treated with HRF to undergo a second set of strut adjustments after the initial correction is completed to fine-tune the final alignment. Depending on the pathology and treatment plans, patients may require HRF for extended durations, usually 3–12 months. Complications with the procedure may also extend treatment times and are challenging for the patients [10–12].

There are only a few studies documenting patient experience over 1- or 2-years of HRF use, in part due to its relative uncommon utilization, and a wide variety of indications [10, 12–15]. We designed two studies to analyze the intra- and postoperative experience of patients with HRF application. This current study focuses on the post-operative period following HRF application and is designed to analyze the clinical presentation, risks, outcomes, and payer costs of patients requiring application of an HRF, from the day of, and up to, 2 years post HRF application, based on initial etiology.

## Methods

### Data sources

Data used in this analysis were obtained from the IBM Marketscan® Research Databases. These databases comprise enrollment, demographics, complete inpatient and outpatient medical information, and outpatient pharmacy claims data. The commercial database includes information for individuals who are under the age of 65 and are the primary insured or a spouse or dependent thereof. A total of 155 million distinct patients with average age of 31 are covered in the database.

Ethics approval from an Institutional Review Board and Informed Consent were not required for this study as it used data from an anonymous, de-identified, administrative claims database compliant with the Health Insurance Portability and Accountability Act of 1996.

### Patient population

All patients less than 65 years of age with HRF application (with a common procedural code (CPT) 20,696: application of multiplane fixation system with stereotactic computer-assisted) were identified in the IBM Marketscan® Commercial Claims database between 2007 and February 1st, 2019 and were followed for up to two years post-surgery. The date of surgical application of the frame was defined as the “index” date. Patients were categorized based on the following categories, which were developed based on primary and secondary diagnostic codes associated with the index procedure.

1. Congenital deformity: patients less than 17 years of age, with at least one diagnosis of congenital deformity and no other diagnoses of osteomyelitis, nonunion or fracture.
2. Complex congenital deformity: patients less than 17 years of age, with a congenital deformity, and concurrent diagnoses of fracture or infection or nonunion.
3. Acquired deformity: patients 17 years or older, with a diagnosis of deformity and no diagnoses of deep infection or nonunion or fracture or arthropathy.

The following categories included patients of all ages but pediatric (defined as < 17) and adult (defined as ≥17) patients were analyzed separately:

4. Fracture: patients with a diagnosis of acute fracture and no concurrent diagnosis of deep infection or nonunion or other sequelae, suggesting prior unresolved fracture pathology.
5. Deep infection, with or without nonunion: patients with deep infection diagnoses (osteomyelitis or infection due to internal fixation or pyogenic arthritis), with or without nonunion diagnoses. Pediatric vs adult patients were further analyzed separately. Pediatric patients with deep infection and congenital deformity were categorized as complex congenital deformity cases, as described above.
6. Non-union without deep infection included patients with non-union diagnoses but no deep infection diagnoses.
7. Arthropathy: Patients with a diagnosis of arthropathy and none of the other diagnoses listed above (deformity, fracture, infection, or non-union) were included in this cohort.

**Fig. 1:**
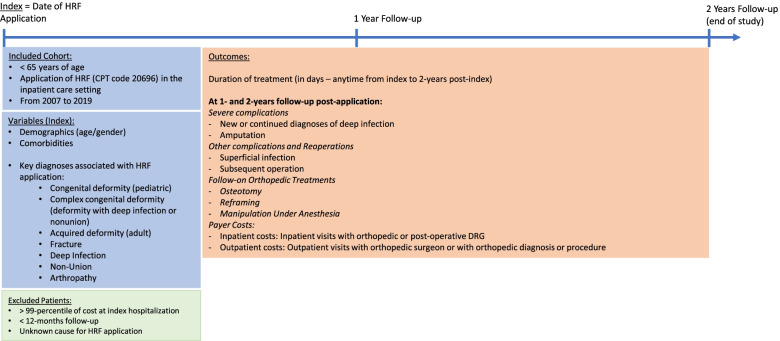
Diagrammatic representation of the study design

Patients that did not meet any of the defined categories were excluded, as the exact cause for the use of the HRF could not be determined. In the per-category analyses: groups containing less than 30 patients were not analyzed separately as statistical analyses were not meaningful in such small sample sizes. Figure 1 provides a diagrammatic representation of the study design.

### Study measures

#### Variables: baseline demographic and clinical characteristics

Patient demographics that were evaluated included age, sex, and calendar year at time of the index hospitalization. Baseline comorbidity (i.e., comorbid conditions present before, and up to the day of application of the frames, other than those defined by etiology) was assessed using the Elixhauser Comorbidity Index, an aggregate measure of comorbidity created by using 31 dimensions associated with chronic disease (e.g., heart disease, cancer) and overall health conditions. Each of the 31 disease states was also analyzed separately. Higher values on Elixhauser are associated with greater comorbidity. Prior research has shown that increasing Elixhauser scores are associated with increased risk of mortality; Elixhauser scores are therefore a good measure of overall health status and risks.^31^

#### Outcomes

The following outcomes were identified for all patients: 1) Overall healthcare resource utilization (HCU) and costs, 2) deep infection defined as presence of osteomyelitis or bone infection at 1- and 2-years post-index; 3) superficial infection; 4) New operations/reoperations, and amputations at 1- and 2-years post-index, and 5) Additional orthopedic procedures, suggesting ongoing orthopedic interventions after frame removal (Manipulation under anesthesia (MUA), osteotomy, or application of a new HRF).

#### Healthcare resource use, complications, and insurance payments over the follow-up period

Payments for healthcare services and counts of key orthopedic procedures, including visits for imaging and physical therapy, were analyzed based on CPT codes and compared across groups. Duration of treatment was estimated by calculating the duration from index to HRF removal, identified in the database with CPT 20694: removal, under anesthesia, of external fixation system. For some patients, this code was not available. In cases where no visit with a CPT 20694 was identified in a patient’s records, an imputed duration was estimated based on the average duration of the treatment for patients in the same diagnostic category. Cases with no CPT 20694 code were not included in the final estimation of treatment duration; the imputed durations were simply used as a starting point to evaluate the rate of post-HRF removal treatments and complications.

#### Deep infection

Deep infection was analyzed differently for patients that had already deep infection at index versus those that did not. For patients that did not have a deep infection at index, deep infection was defined as having at least 1 diagnosis of deep infection from one day post-index to one- or two years post-index. For patients with existing deep infection at index, (continued, unresolved) deep infection was defined as having at least 1 diagnosis of deep infection from day 91 after index to one- or two-years post index. For these patients, the 90-day window was used to distinguish deep infection at index from a continued, unresolved deep infection.

#### Superficial infection

Superficial infection was defined as having at least 1 diagnosis indicative of skin/wound infection from one day post-index to one-year post-index was required. This comorbidity was only followed to 1 year post-index as it was only relevant while the frames were in place.

#### New operations/reoperations and amputation

New operations/reoperations were defined as readmission with an ICD or CPT code indicative of long bone surgery involving an external fixator. Amputation was defined as the presence of an ICD procedure code specific for amputation on the index limb from one day post-discharge to up to 2 years post-index. Amputation was defined as having an inpatient visit with an amputation code within the follow-up period.

#### Additional orthopedic procedures

Post-frame removal care outcomes were defined as the following: MUA was identified in patients with a CPT code for MUA from one day post-frame removal to one-year post. Osteotomy and/or new frame application were similarly identified for all patients with appropriate CPT or ICD codes after the frame removal and up to 1 year.

All outpatient visits in the 12 months following the HRF application were further queried as follows: visits to an orthopedic surgeon or visits with a CPT code indicative of orthopedic care, wound care, physical therapy, or imaging of lower extremities or lower leg anesthesia were included. All inpatient visits with a Diagnostic Related Group (DRG) indicative of orthopedic care or aftercare were included. For all visits, count of services as well as inflation-adjusted payments for inpatient and outpatient services, from index to 12-months post-index, were identified. All payments were adjusted for inflation to the 2020 consumer-price index.

### Statistical analyses

All study variables were analyzed descriptively. Counts and proportions (dichotomous variables) and mean and standard deviation (SD) (continuous variables) were provided. Estimate of payments and duration of HRF application adjusted for age, comorbidity, and gender – for each diagnostic category of patients (with *N* > 30) – were generated using generalized linear models with log link function and gamma distribution. For payment estimates: analyses were only performed on patients with reported index payments > $1000 (to eliminate cases with missing reported payments). For the duration of HRF application estimates: models only included patients with a CPT 20694 (device removal code) in the post-index period. Estimates of healthcare resource utilization were generated from Poisson models, adjusting for age, gender, and comorbidity. Finally, adjusted rate estimates for post-frame removal adverse events (within 12 months of frame removal: re-framing, MUA, and new osteotomies) and overall complications (up to 2 years post-index: new surgeries involving external frames, deep infection, superficial infection, amputation) were generated using Poisson regressions with log links. Logistic regression models were built to evaluate risk factors for amputation and deep infection, up to 2 years post-index. For these models, the variable selection was performed using stepwise regression (R package: MASS – function stepAIC). All statistical analyses were performed in R (version 4.0.3) using the Rstudio interface (version 1.4.1103).

## Results

A total of 1150 patients were identified. Of these, 11 patients had a total index hospital payment exceeding US $700 K or 45 days and thus were above the 1-percentile in healthcare utilization. These patients were excluded as they represented outliers and would skew overall findings, although these patients do represent an important category from a clinical and economic standpoint. In addition, 424 did not have a full 12-months follow-up and an additional 24 could not be categorized based on the diagnostic groups defined above, the reason for which they had an HRF system applied was therefore unknown. These patients were also excluded.

### Baseline demographic and clinical characteristics

A total of 695 patients with at least 12 months of follow-up were therefore included in our study. Their demographic and comorbid scores are shown in Table 1.

**Table 1:** Baseline demographic information and comorbidity indices of HRF patient cohort

Variables	Overall		Patient Group			
			Younger than 17		17 and Above	
	N	%	N	%	N	%
All	695	1	228		467	
Female	286	41%	93	41%	193	41%
Patients with 2 year follow-up	443	64%	158	69%	285	61%
Age (mean (SD))	33.07 (19.44)		11.34 (3.91)		43.69 (14.55)	
***Age category***						
Less than 17	228	33%	228	100%	0	
17 to 25	85	12%	0		85	18%
26 to 45	131	19%	0		131	28%
46 to 64	251	36%	0		251	54%
***Etiology***						
Congenital Deformity	168	24%	168	74%	0	
With fracture or sequelas from prior fracture	12	2%	12	5%	0	
Deep Infection	68		3	1%	68	14%
With non-union			0		41	9%
Fracture	219	32%	35	15%	184	39%
Non-Union	86	12%	5	2%	81	17%
Other Sequelas from Prior Fractures	6	1%	5	2%	1	0%
Acquired Deformity	103	15%	0		103	22%
Arthropathy	31	4%	0		31	7%
Average Elixhauser Score (mean (SD))	1.15 (1.71)		0.29 (0.60)		1.57 (1.91)	
***Elixhauser Score Category***						
less than 1	359	52%	177	78%	182	39%
1 or 2	217	31%	48	21%	169	36%
3 or 4	85	12%	3	1%	82	18%
5 or greater	34	5%	0		34	7%

In the pediatric group, the majority (73.7%) had congenital deformity without infection or other severe complications. An additional 15.4% were fracture cases. Complex congenital deformity (congenital deformity with fractures and/or deep infection), deep infection, nonunion and other sequelae from prior fractures accounted for the remaining 10.9%.

In the adult group, deformity alone only accounted for 22.1% of cases. The majority of cases had fractures (39.4%) or infections, nonunions or other sequelae from prior fractures (38.5%). Comorbid scores differed across age groups: in the pediatric group, the vast majority (77.6%) had no comorbidities. Only 1.3% of cases had 3 or 4 comorbidities. In the adult group, 24.8% of cases had 3 or more comorbid conditions, and only 39% of cases had no comorbidities.

Analyses of comorbidities are shown in Table 2.

**Table 2:** Key baseline comorbidities at the time of HRF application. 2A: Key comorbidities by age category (adult: 17 years and above, vs pediatric: less than 17 years). 2B: Key comorbidities by diagnostic category

2A - Comorbidity by Patient Age Group					
**Variables**	**Overall**	**Adults**	**Pediatric**		
**All Hypertension (with or without complications)**	24.5%	36.2%	0.4%		
**All Diabetes (with or without complications)**	19.6%	28.9%	0.4%		
*** Complicated Diabetes***	*7.2%*	*10.7%*	*0.0%*		
**Depression**	11.4%	14.6%	4.8%		
**Chronic pulmonary disease (including asthma)**	9.2%	10.3%	7.0%		
**Obesity**	8.2%	10.7%	3.1%		
**Cardiac arrhythmias**	7.2%	9.9%	1.8%		
**Hypothyroidism**	5.5%	7.7%	0.9%		
**Fluid and electrolyte disorders**	4.9%	6.9%	0.9%		
**Peripheral vascular disorders**	4.0%	5.8%	0.4%		
2B - Comorbidity by Diagnostic Group in Adults	**Acquired Deformity**	**Arthropathy**	**Deep Infection**	**Fracture**	**Nonunion**
**Age (mean, standard deviation)**	**34.32 (14.27)**	**53.55 (8.88)**	**46.24 (13.66)**	**43.84 (14.11)**	**49.26 (12.08)**
**All Hypertension (with or without complications)**	20.4%	58.1%	47.8%	30.4%	51.9%
**All Diabetes (with or without complications)**	15.5%	41.9%	56.7%	22.8%	32.1%
*** Complicated Diabetes***	*5.8%*	*16.1%*	*26.9%*	*6.5%*	*11.1%*
**Depression**	13.6%	16.1%	22.4%	11.4%	16.0%
**Chronic pulmonary disease (including asthma)**	17.5%	9.7%	7.5%	6.0%	13.6%
**Obesity**	10.7%	16.1%	13.4%	8.7%	11.1%
**Cardiac arrhythmias**	2.9%	12.9%	13.4%	9.8%	14.8%
**Hypothyroidism**	1.0%	3.2%	13.4%	8.2%	12.3%
**Fluid and electrolyte disorders**	3.9%	3.2%	16.4%	6.0%	6.2%
**Peripheral vascular disorders**	4.9%	19.4%	7.5%	1.6%	9.9%
2B - Comorbidity by Diagnostic Group in Pediatric Patients	**Congenital Deformity**	**Fracture**			
**Age (mean, standard deviation)**	**10.51 (4.06)**	**13.77 (2.44)**			
**All Hypertension (with or without complications)**		2.9%			
**All Diabetes (with or without complications)**		2.9%			
*** Complicated Diabetes***					
**Depression**	1.8%	11.4%			
**Chronic pulmonary disease (including asthma)**	4.8%	17.1%			
**Obesity**	1.8%	5.7%			
**Cardiac arrhythmias**	2.4%				
**Hypothyroidism**	0.6%				
**Fluid and electrolyte disorders**	0.6%	2.9%			
**Peripheral vascular disorders**	0.6%				

Hypertension, diabetes, depression, chronic pulmonary disease, and obesity were the top 5 comorbidities. However, the majority of these comorbidities were predominantly present in adults. Hypertension and diabetes affected 36.1 and 29.0% of adults, respectively. The main pediatric comorbidity was chronic pulmonary disease (primarily asthma), depression, and obesity, at 7.0, 4.8, and 3.1%, respectively. These percentages were far lower than in adults (10.3, 14.6, and 10.7%, respectively). A closer analysis by diagnostic group further highlighted the differences between patient groups; some of these differences in comorbidities might be explained by average age differences. For adults: patients with acquired deformity were young relative to other patients (34 years vs > 43 for all other groups) and presented with few comorbidities, as may be expected from relatively healthy young individuals. The arthropathy group was the oldest and had the highest prevalence of hypertension (58.1%), obesity (16.1%), and vascular disorders (19.4%). Diabetes was the main comorbidity in patients with deep infection (56.7%), and 26.9% of all deep infection patients had diabetes with complications. Deep infection patients also presented with high rates of depression (22.4%). For pediatric cases: pulmonary disease/asthma, obesity, and depression were observed in the complex deformity group at a rate of 16.7, 8.3, and 16.7%, respectively, and in the fracture group (17.1, 5.7, and 11.4%, respectively).

### Healthcare resource use over the follow-up period

Healthcare resource utilization is shown in Table 3. Six patients had incomplete payment information and were removed from the payment analysis. Payments for the index surgery, during which the HRF was applied, ranged from $39,250 for patients with arthropathy to $75,350 for patients with deep infection. Means with confidence intervals as obtained from the GLM models are shown in Table 3. In the 12 months post-index, the adult arthropathy group had a relatively low mean post-index payment (approximate mean: $14,350) whereas all other patient groups had averages post-index payments exceeding $30,000. Patients with deep infection and adults with fractures presented with the highest post-index payments, at approximately $40,340 and $43,108, respectively. The reason for these very high post-index payments was further analyzed by looking at the duration of treatment and count of treatments related to orthopedic care.

**Table 3:** Resource utilization associated with, and treatment duration of, HRF. 3A: Inflation-adjusted payments for index and orthopedic care in the 12 months post-index. 3B: Duration of treatment and frequency of physical therapy and imaging visits post-index

**3A: Payments by Index Diagnostic Category**	**Index Payment**		**Post-Index 12-Month Payments**
**Older than 17 - Acquired Deformity**	$52,621.32 ($44,118.08–$65,184.96)		$35,670.52 ($27,587.92–$50,451.63)
**Older than 17 – Arthropathy**	$39,248.23 ($27,733.76–$67,111.51)		$14,354.50 ($9340.97–$30,984.81)
**Older than 17 - Deep Infection**	$75,347.00 ($59,274.92–$103,377.16)		$40,336.51 ($29,541.45–$63,564.19)
**Older than 17 – Fracture**	$70,050.28 ($60,024.18–$84,097.44)		$43,108.13 ($33,919.26–$59,125.47)
**Older than 17 – Non Union No infection**	$48,623.22 ($39,166.01–$64,101.47)		$38,293.43 ($28,040.84–$60,364.57)
**Younger than 17 – Congenital Deformity**	$58,699.77 ($49,132.44–$72,894.10)		$35,986.88 ($27,363.52–$52,546.37)
**Younger than 17 – Fracture**	$58,395.84 ($43,801.64–$87,574.72)		$30,403.12 ($20,092.63–$62,448.29)
**3B: Duration and Healthcare Utilization, by Index Diagnostic Category**	**Duration of Treatment (in Days)**	**Count of Post-Index Outpatient Physical Therapy Sessions**	**Count of Post-Index Outpatient Imaging Procedures**
**Older than 17 - Acquired Deformity**	127.98 (114.50–145.04)	18.75 (17.82–19.72)	9.81 (9.17–10.50)
**Older than 17 – Arthropathy**	96.41 (68.81–160.97)	11.52 (10.14–13.09)	4.65 (3.90–5.55)
**Older than 17 - Deep Infection**	174.11 (148.98–209.46)	8.04 (7.30–8.86)	12.65 (11.72–13.65)
**Older than 17 – Fracture**	128.87 (116.85–143.66)	20.14 (19.23–21.08)	9.59 (9.02–10.19)
**Older than 17 – Non Union No infection**	142.98 (124.54–167.84)	10.49 (9.65–11.41)	12.21 (11.30–13.19)
**Younger than 17 – Congenital Deformity**	149.50 (132.42–171.63)	22.26 (21.10–23.49)	8.94 (8.30–9.62)
**Younger than 17 – Fracture**	114.37 (94.64–144.48)	16.78 (15.42–18.26)	8.68 (7.68–9.82)

The exact duration of the treatment was available (through the presence of CPT 20694 with corresponding timestamp) for 85.8% of patients. These data contributed to the modeling of the treatment duration by index diagnostic category. Duration of care and healthcare utilization summary findings are shown in Table 3. Arthropathy patients had the shortest duration of treatment, approximately 3 months (96 days). Young patients treated for fractures had an average treatment slightly lower than 4 months, for all other patients, the average duration exceeded 4 months, and for deep infection, it nearly averaged 6 months (174 days). Counts of outpatient physical therapy (PT) and imaging are provided as an example of the extensive treatments endured by HRF patients. Young patients treated for deformity had the highest overall PT utilization, with an average of 22 visits in 12 months, nearly twice a month. Adult fracture and deformity cases also attended a significant average number of PT sessions in the 12 months post-application (approximately 20 and 18, respectively). Counts of postoperative imaging claims were lowest for arthropathy (4.65) but exceeded 12 events per year for patients with deep infection and nonunion. The pediatric fracture and deformity groups experienced approximately 8–9 imaging visits during the treatment.

### Postoperative complications and reoperations over the follow-up period

The risks for superficial infection, deep infection, amputation, and additional surgery/reoperations during the entire treatment time and up to 2 years post-index were estimated. In addition, the following risks immediately following removal of the frame were analyzed: re-application of a new frame, new osteotomy procedure, indicative of further requirement for correction, new manipulation under anesthesia (MUA).

Th risks of infection, amputation, and subsequent surgical procedures, for each diagnostic category, are shown in Tables 4 and 5. The risks for superficial, and deep infection, amputation and additional surgery/reoperation are shown in Table 4A. Superficial infection was only measured up to 1-year post-application as it was most relevant during the period in which the frame was in place. Adult arthropathy and acquired deformity patients were at lower risk for superficial infection (3 and 14%, respectively) whereas most other patients had a risk of approximately 20%, with the highest being observed in patients with deep infection (39%). For all diagnostic categories, however, the confidence intervals for superficial infection risks were broad and overlapped.

**Table 4:** Risk for infections and additional surgery following application of the HRF, within 1 year (for superficial infection) and 2 years (for deep infection, amputation and additional surgery)

Diagnostic Categories at Time of Index	Superficial Infection During HRF Application (up to 12 months post-index)	New Deep Infection	Amputation	Additional Surgery
**Older than 17 – Acquired Deformity**	13.6% (7.6–24.4%)	10.5% (4.7–23.4%)	0.0%	18.1% (8.7–37.6%)
**Older than 17 – Arthropathy**	2.8% (0.4–20.5%)	5.2% (0.7–40.5%)	0.0%	3.7% (0.4–31.3%)
**Older than 17 - Deep Infection**	39.1% (25.0–61.0%)	57.8% (33.6–99.4%)	8.9% (3.3–23.9%)	7.6% (2.2–26.0%)
**Older than 17 - Fracture**	21.2% (14.0–32.0%)	6.8% (3.0–15.7%)	2.7% (0.9–7.7%)	6.0% (2.2–16.1%)
**Older than 17 – Nonunion No Infection**	20.4% (11.7–35.4%)	20.1% (9.4–43.0%)	5.0% (1.6–15.3%)	10.3% (3.5–30.1%)
**Younger than 17 – Congenital Deformity**	18.5% (10.4–33.0%)	3.8% (1.4–10.6%)	0.0%	17.4% (7.3–41.6%)
**Younger than 17 – Fracture**	21.2% (8.3–54.2%)	7.3% (1.7–32.0%)	0.0%	10.2% (2.2–48.2%)

**Table 5:** Post-index Outcomes in Patients with Deep Infection, with and without Nonunion at Index

		Index Diagnostic Category: Deep Infection without Nonunion	Index Diagnostic Category: Deep Infection with Nonunion
**HCU**	**Post-index 12 Months Payment**	$32,146 ($21,067–$67,806)	$44,247 ($30,078–$83,649)
	**Count of Post-Index Outpatient Imaging**	11.5 (10.2–12.9)	13.3 (12.1–14.6)
	**Count of Post-index Physical Therapy Visits**	7.1 (6.1–8.3)	8.5 (7.5–9.6)
	**Duration of Treatment (days)**	144.0 (115.7–190.4)	190.0 (157.1–240.3)
**Outcomes (2 years post index)**	**Superficial infection**	22.1% (9.8–50.1%)	51.4% (31.4–84.1%)
	**New Diagnoses of Deep Infection**	44.6% (24.1–82.4%)	60.2% (36.4–99.7%)
	**New Surgery/Reoperation**	7.6% (1.9–31.0%)	12.2% (4.7–31.5%)
	**Amputation**	6.8% (1.8–25.6%)	5.5% (1.6–19.1%)

The presence of post-index deep infection was particularly high for patients in the deep infection diagnostic category, reaching 58% although for these patients, deep infection was defined as a new diagnosis of deep infection *after* 90 days post-index (to avoid counting diagnosis related to a history of deep infection). For other adults, patients with arthropathy were again at the lowest risk (5%), followed by patients with deformity (10%) and fractures (7%). In pediatric cases, deep infection was observed in 7% fracture cases and 5% deformity correction cases.

The risk for repeat, new surgery was most prevalent in deformity correction patients (for adults: 18%, for pediatric: 17%). Fracture and arthropathy patients had low rates of new surgery (less than 7%). Non-union and deep infection patients had a risk for new surgery ranging from 7 to 10%.

Amputation was only observed in patients with deep infection (8.9, 95%CI: 3.2–23.9%), nonunion (5.0, 95%CI: 1.6–15.4%) and fracture (2.7, 95%CI: 0.9–7.6%).

The risks for re-treatments were as follows: MUA and osteotomy were excessively rare (lower than 1% in all groups). Re-application of the frame was less than 4% except for adult deformity cases (16.8, 95%CI: 9.3–30.2%) and pediatric deformity cases (10.9, 4.5–26.2%).

### Analysis of deep infection patients: with and without nonunion

Deep infection patients with and without nonunion did not differ in terms of demographics or comorbidities. They also did not differ in terms of index costs. For this reason, only aggregate results are shown for all deep infection patients. However, a closer analysis of the post-index period revealed that for all post-index care and complications except amputation, patients with deep infection *with* nonunion at index had payments, number of visits, and risks for infection and new surgeries/reoperations. The details of the post-index outcomes, for patients with deep infection with and without nonunion, are shown in Table 5.

### Risks for complications

**Fig. 2:**
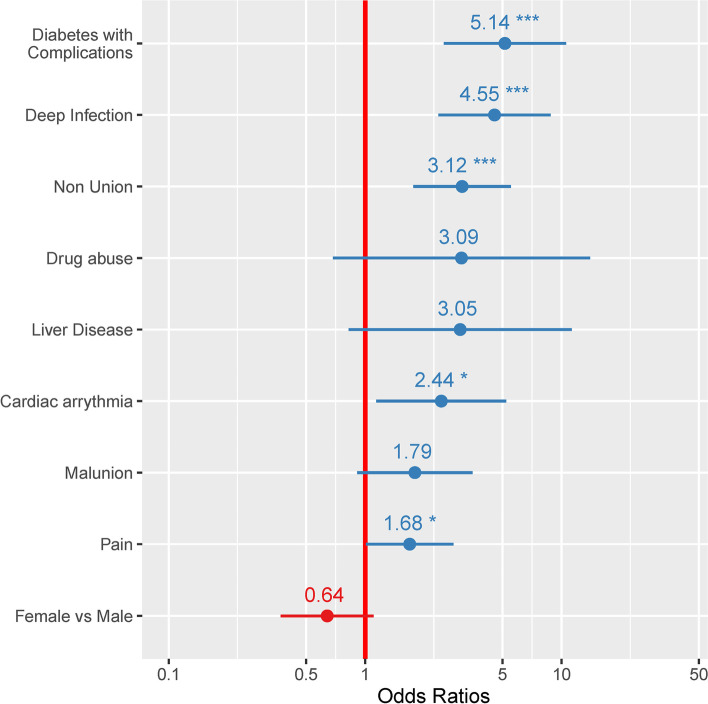
Forest plot of output from the logistic regression model evaluating odds for deep infection in the 2-year post-index. The variables identified in the final model, with the corresponding odds ratios and confidence intervals, are shown in the figure. Diabetes was the main comorbidity predictive of post-index deep infection. Patients in the deep infection or nonunion diagnostic category were also at high risk of deep infection (continued or new). Females were at slightly lower risk for deep infection, although these odds were not significant

**Fig. 3:**
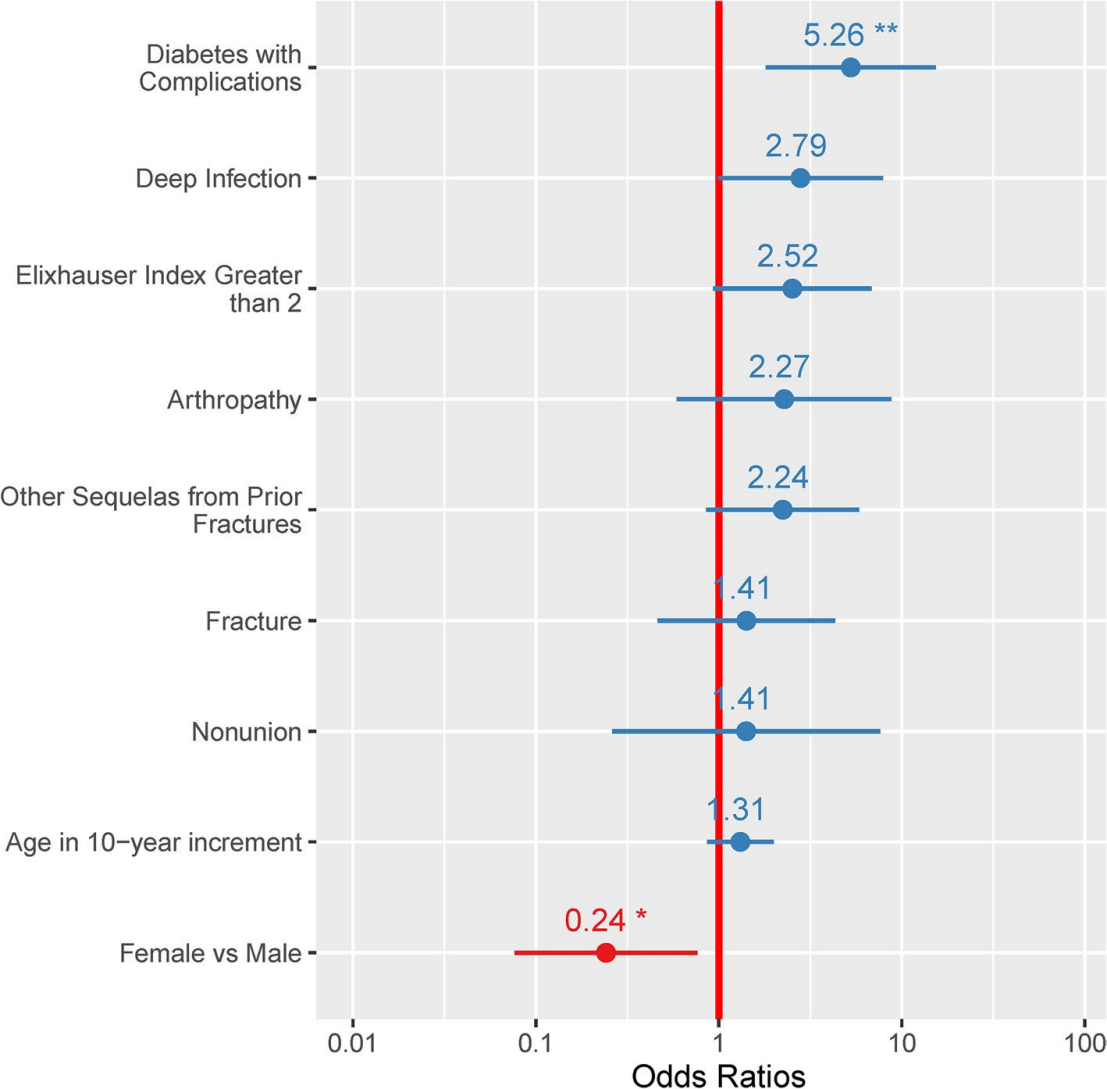
Forest plot of output from the logistic regression model evaluating odds for amputation in the 2-year post-index. This model did not include any of the pediatric cases or adult cases treated for deformity correction only as no amputation was observed in those groups. For all other groups, diabetes with complications was the greatest significant risk factor for amputation. None of the other variables in the final model were significantly associated with increased odds for amputation

Risk for deep infection and amputation were modeled using logistic regression, as described above. Figures 2 and 3 show forest plots of the logistic regression outputs, with the key variables retained in the final models. Diabetes was the main comorbidity predictive of deep infection (OR: 5.14, 95%CI: 2.50–10.54) and amputation (OR: 5.26, 95%CI: 1.79–15.51). For deep infection: patients with an index diagnostic category of deep infection and/or nonunion were at high risk of new or continued deep infection. Females were at slightly lower risk for deep infection, although these odds were not significant. For amputation, the models did not include any of the pediatric cases or adult cases treated for deformity correction as no amputation was observed in those groups. For all other groups, diabetes with complications was the greatest significant risk factor for amputation. None of the other variables in the final model were significantly associated with increased odds for amputation.

## Discussion

Findings from this longitudinal analysis highlight the significant heterogeneity in comorbidities and index clinical presentation of patients treated with HRF, as well as the long-term intensity of care, treatment, and financial impact of HRF treatments. Patients treated for deformity have fewer comorbidities, on average longer treatment duration but also better prognosis and low risk for deep infection and amputation compared to patients treated for deep infection. In comparison, patients with deep infection at the time of HRF application had a higher risk of amputation than any other patient group. Patients with a diagnosis of arthropathy generally had the application of HRF to undergo joint distraction and deserve special mention. This group was the oldest with the expected high rates of hypertension, vascular disease, and obesity. They had the shortest duration of time in frame and the lowest payments for both the index procedure as well as payments for the postoperative follow-up period, compared to all other patient groups.

Diabetes with complications was a significant risk factor for deep infection and amputation, this finding is consistent with other studies and highlights the importance of diabetes control in patients undergoing orthopedic care [16, 17]. Using existing diagnostic codes from claims databases and methods described elsewhere [18], presence of complicated diabetes (i.e. diabetes with organ involvement) can be analyzed for all patients, but exact A1c values are not available, it is therefore unfortunately not possible to determine the extent of diabetes with more accuracy.

This difference in risk and outcomes across populations treated with HRF suggests that clinical studies evaluating the safety and efficacy of HRF cannot be generalizable beyond the exact study populations and diagnoses under investigation.

Five prior longitudinal studies on HRF systems were identified in the published literature [10, 12–15]. The five studies are small relative to the current study and are not easily generalizable [10, 12–15]. Four of the prior studies were retrospective patient chart reviews with sample sizes ranging from 16 to 102 patients [12–15] and one of the prior studies was a patient survey (sample size of 14 patients) [10]. The overall rate of superficial infection in the current study was 20.5% and the rate of deep infection was 17.8%, whereas in other published studies the definitions of infection varied, and the rates of infection ranged from 2.0 to 64.0% [10, 12–14].. Prior studies focusing on pediatric cases typically report very low deep infection rates, as observed in our study [13, 14]. Our overall risk of infection is therefore a reflection of the fact that our analysis included the full spectrum of patients, from the generally healthy pediatric cases presenting for deformity correction all the way to adult patients with an infected nonunion with bone loss. The 7.8% rate of reoperation in the current study was lower than that reported in prior published studies (range 12.5–14.3%) [10, 12].

It is important to note that in many treatment plans for complex limb reconstruction, additional surgery is planned such as an HRF modification, additional debridement, flap coverage, or a bone grafting procedure. But since the indications for these additional surgical procedures were not obtainable, differentiation between planned and unplanned surgery post-index procedure was not possible based on CPT codes.

Rates of deep infection were – expectedly – highest in patients that already presented with a deep infection at index. For these patients, we estimated new infections as the presence of diagnoses of infections after a 3-month window following the index procedure. This window was designed to exclude cases where a visit may have triggered a diagnosis of deep infection simply due to the patient’s history. A history of deep infection could have triggered new inaccurate codes of infection in the 3-months post-index. These inaccuracies would inflate our estimates.

This study also characterized the difficulty in treating patients with deep infection with concurrent nonunion. These patients had the highest average duration of treatment (190 days) and post-index payments (approximately $44 K). Sixty percent of them had continued infections between 90 and 730 days post index, and 12% required additional surgery. The difficulty in treating these patients has been described thoroughly by others [19–22].It is important to note that the use of Ilizarov techniques and external fixators has been credited for reducing the failure rate of these cases from more than 30% to less than 10%, as observed in our cohort [23]. Our findings also identified the fact that these patients are more likely to be associated with high levels of comorbidities, and these comorbid medical conditions must be taken into consideration when designing a treatment plan for this challenging population.

The main limitation of this study is that it used administrative claims data, which are not collected specifically for research purposes. Claims data are at risk of having clerical inaccuracies, recording bias secondary to financial incentives, and temporal changes in billing codes [24, 25]. In our case, this was particularly true for analyses of reoperation and deep infection, as noted above. However, administrative claims databases have advantages of very large sample sizes and good longer-term follow-up of patients. The study findings may also be limited in their generalizability and may not apply to patients without commercial health insurance and to patients outside of the US. An additional limitation is related to our calculation of payments: Insurance payments were calculated but do not account for patient out-of-pocket costs or lost productivity. As the medical community looks at ways to improve outcomes, the impact on patients and caregivers needs to be included. Despite these limitations, this study provides an informative overview of the entire clinical experience, from time of frame application to 2-years post-index, of a relatively large population of patients treated with HRF, characterized by etiology.

## Conclusions

Findings from this longitudinal analysis demonstrate the significant heterogeneity in patients treated with HRF, and the wide range in treatment intensity, payment and outcomes. Risks for deep infection and amputation were primarily linked to presence of complicated diabetes at the time of frame application and reflect the difficulty of limb salvage in this population. Our findings suggest a need for careful management of comorbid chronic conditions in patients requiring HRF for orthopedic care.

## Data Availability

The data for these analyses were made available to the authors by third-party licenses from MarketScan (https://www.ibm.com/watson-health/products), a data provider in the US. Under the licensing agreement, the authors cannot provide raw data themselves. Other researchers could access the data by purchase through MarketScan, and the inclusion criteria specified in the Methods section would allow them to identify the same cohort of patients we used for these analyses.

## Abbreviations

CPT: Common Procedural Terminology
DRG: Diagnostic Related Group
HCU: Health Care Resource Utilization
HRF: Hexapod Ring Fixation
ICD: International Classification of Disease
LOS: Length of Stay
MUA: Manipulation Under Anesthesia
ORT: Operating Room Time
SD: Standard Deviation
SNF: Skilled Nursing Facility
